# Supplementary material for: Contextualizing future maternal RSV vaccination acceptance and trust among pregnant and lactating women in Kenya: A latent class analysis
Source: PLOS Glob Public Health. 2025 Aug 28;5(8):e0004505. doi: 10.1371/journal.pgph.0004505 (PMC12393705; doi:10.1371/journal.pgph.0004505)
Supplement: S1 Table — aResponses were dichotomized to strongly agree/agree and disagree/strongly disagree/don’t know for analysis; bRSV = Respiratory syncytial virus. (DOCX) [file pgph.0004505.s001.docx]

| **Group** | **Survey item** | **Response options^a^** |
| --- | --- | --- |
| Perceived prevalence | The majority of babies <2 years old get RSV.^b^ | Strongly agree  Agree  Disagree  Strongly disagree  Don’t know |
| Perceived risk | I worry that my baby could get RSV. | Strongly agree  Agree  Disagree  Strongly disagree  Don’t know |
|  | I believe RSV is dangerous for babies. |  |
|  | I believe RSV is dangerous for pregnant women or women who have recently given birth. |  |
| Social norms | If there was a Ministry of Health approved maternal vaccine for RSV, the majority of my pregnant friends and family would get it. | Strongly agree  Agree  Disagree  Strongly disagree |
|  | If there was an approved maternal vaccine for RSV, the majority of my friends and family would encourage me to get it. |  |
| Self-efficacy | I have some control over whether or not I get vaccines during my pregnancy. | Strongly agree  Agree  Disagree  Strongly disagree |
| Barriers | If I need to visit a health facility for an appointment or a vaccine, I can easily go to that health facility. | Strongly agree  Agree  Disagree  Strongly disagree |
| Safety | I am confident that vaccines recommended for me during pregnancy are safe for me. | Strongly agree  Agree  Disagree  Strongly disagree |
|  | I am confident that vaccines recommended for me during pregnancy are safe for my baby. |  |
| Benefits | If a new vaccine were approved for use among pregnant women, I trust that the vaccine would protect me. | Strongly agree  Agree  Disagree  Strongly disagree |
|  | If a new vaccine were approved for use among pregnant women, I trust that the vaccine would protect the fetus. |  |
