# Supplementary material for: Contextualizing future maternal RSV vaccination acceptance and trust among pregnant and lactating women in Kenya: A latent class analysis
Source: PLOS Glob Public Health. 2025 Aug 28;5(8):e0004505. doi: 10.1371/journal.pgph.0004505 (PMC12393705; doi:10.1371/journal.pgph.0004505)
Supplement: S1 Checklist — (DOCX) [file pgph.0004505.s002.docx]

Inclusivity in global research

PLOS’ policy on inclusivity in global research aims to improve transparency in the reporting of research performed outside of researchers’ own country or community and ensures that PLOS publications reporting global research adhere to high standards for research ethics and authorship. Authors of relevant research articles may be asked to complete the questionnaire below, which outlines ethical, cultural, and scientific considerations specific to inclusivity in global research. This questionnaire may be requested when researchers have travelled to a different country to conduct research, if research uses samples collected in another country, research with Indigenous populations or their lands, or if research is on cultural artefacts. Researchers travelling to another country solely to use laboratory equipment will not normally be required to complete the questionnaire. However, the questionnaire can be requested at the journal’s discretion for any submission – if you have been requested to complete this questionnaire by the PLOS journal you submitted to, please do so.

Please complete the questionnaire below and include this as a Supporting Information file with your manuscript. Note that if your paper is accepted for publication, this checklist will be published with your article in the supporting information files. Please ensure that you reference the checklist in the main body of your manuscript. We suggest adding a subsection ‘Inclusivity in global research’ to your Methods section and adding the following sentence: “Additional information regarding the ethical, cultural, and scientific considerations specific to inclusivity in global research is included in the Supporting Information (SX Checklist)”

The questions have been designed to be applicable to a wide range of study types, and there are subsections for both human subjects research and non-human subjects research. If any of the questions are not relevant to your research please mark them as “N/A” as appropriate.

**Ethical considerations, permits and authorship**

*This section is applicable to all research types.*

Provide details as to who granted permissions and/or consent for the study to take place in the Methods section of your manuscript. This should include the names of **all** ethics boards, governmental organizations, community leaders or other bodies that provided approval for the study. If individuals provided approval refer to these people by their role or title but do not list their name(s).

Reported on page number: 11

If there were any deviations from the study protocol after approval was obtained please provide details of these changes in the Methods section of your manuscript.

Reported on page number: n/a

Did this study involve local collaborators that are residents of the country where the research was conducted or members of the community studied? If you do not have any authors from said communities, please provide an explanation for this below.

The MIRI study involved local collaborators who are residents of the country where the research was conducted. This study was conducted in Kenya as a collaborative effort between scientific, policy, and program institutions in Kenya and the U.S.. This includes team members and co-authors from the Johns Hopkins Bloomberg School of Public Health (USA); Jhpiego (Kenya and USA); National Vaccines and Immunization Program, Ministry of Health (Kenya); and key subject matter experts in Kenya. These individuals were heavily engaged in study activities and are included as authors on this paper. We affirm that all authors meet PLOS criteria for authorship.

Everyone listed as an author should meet PLOS’ criteria for authorship and all individuals who meet these criteria should be included in the author byline, rather than the acknowledgements. For further information please see the journal’s Authorship Policy.

**Human subjects research (e.g. health research, medical research, cross-cultural psychology)**

Did you obtain written informed consent from a representative of the local community or region before the research took place? How did you establish who speaks for the community? Details of written informed consent obtained from study participants should be reported separately in the Methods section of your manuscript.

We obtained oral informed consent from all study participants, in accordance with IRB approvals obtained in Kenya (KEMRI, NACOSTI) and the US (JHSPH IRB); participants marked the survey consent form with an “X” or similar mark; those with low literacy were asked to identify a witness of their own choosing for consent procedures. Consent procedures Prior to the study start, we engaged Ministry of Health officials at the national and sub-national levels (in study counties) and study site leadership (health facility leadership) to introduce study team members; discuss aims and methods, with an emphasis on recruitment and consent processes; address any questions; and affirm their assent for study activities. This included engaging with national health professional bodies (i.e., professional nursing groups, professional pediatrics grops, etc.) to discuss the study, incorporate feedback, and identify potential uses for the resulting data.

How did members of the local community provide input on the aims of the research investigation, its methodology, and its anticipated outcome(s)?

The MIRI study engaged public health officials, representatives of professional medical associations and civil society organizations, and in-country study partners (Jhpiego Kenya and MOH officers) to gather input and refine study aims, methods, and anticipated outcomes. We continued to work closely with these individuals and institutions in Kenya throughout the study, with a particular focus on sharing study learnings with local stakeholders and facilitating their use to inform program and policy discussions in Kenya. Community members (i.e., pregnant and lactating women, family/community members, caregivers, etc. in the two study counties) did not provide direct input on the aims, methods, and outcomes of the study in advance of its conduct.

When engaging with the local community, how did you ensure that the informed consent documents and other materials could be understood by local stakeholders?

Informed consent documents were translated from English to Swahili and validated with native speakers prior to use. Participants were administered consent in their preferred language by trained data collectors. We pilot-tested the consent forms during pre-study data collector training to ensure they were clear and understandable. Data collectors were also trained to provide additional information, explain any concepts, and answer questions raised by potential participants during the consent process.

Will the findings of the research be made available in an understandable format to stakeholders in the community where the study was conducted (e.g. via a presentation, summary report, copies of publications, etc.)? Please provide details of how this will be achieved.

MIRI study results were shared with key stakeholders in both study counties at the culmination of the primary study phase and we continue to share learnings from additional analyses with partners involved in immunization programs, policies, and research in Kenya. Co-authors on this analysis include public health practitioners, decision makers, and health care providers involved in immunization and broader health initiatives in Kenya. While participants themselves will not receive any formal results or resources from this analysis, they will benefit from public health programs that integrate these learnings and are more tailored to their knowledge, attitudes, and beliefs as well as, ideally, the availability of maternal RSV vaccines in Kenya in the near future.

**Non-human subjects research using specimens/ animals collected as part of the study, or those housed in archival collections. Examples include archaeology, paleontology, botany and zoology.**

Did the permission you obtained from a local authority to perform the study include an agreement on access to outputs and benefit sharing? This may include procedures to enable fair distribution of the benefits and resources arising from the research performed. Please include any details of Prior Informed Consent and Benefit Sharing Agreements obtained. These may be required by field-specific regulations, for example the Convention on Biological Diversity (CBD) and the associated Nagoya Protocol.

n/a

If the material used in your study was imported, please A) provide the year it was imported and B) indicate whether permits were obtained to import/export the materials used, C) provide details of any permits obtained. If this information is not available, please indicate this.

n/a

If you used archival specimens, please state how the material used in your study was acquired by the institute it is held in and provide details of any permits obtained for the original excavations/ sample collection. If this information is not available, please indicate this.

n/a

How was the potential cultural significance of the materials collected in your study to local communities considered in your research design? Were Indigenous peoples and/or local researchers and institutions involved with archaeological excavations / collection of specimens? If so, please provide a description of their involvement.

n/a

If your manuscript includes photographs of human remains please indicate whether authors obtained permission from descendants or affiliated cultural communities to do so.

n/a
